# Telephone triage and dispatch of ambulances to patients with suspected and verified acute stroke - a descriptive study

**DOI:** 10.1186/s12873-024-00962-7

**Published:** 2024-03-14

**Authors:** Bjørn Jamtli, Maren Ranhoff Hov, Trine Møgster Jørgensen, Jo Kramer-Johansen, Hege Ihle-Hansen, Else Charlotte Sandset, Håvard Wahl Kongsgård, Camilla Hardeland

**Affiliations:** 1 Faculty of Health Sciences, Oslo Metropolitan University, Oslo, Norway; 2 Prehospital affiliationision, Oslo University Hospital, Oslo, Norway; 3 Department of Neurology, Oslo University Hospital, Oslo, Norway; 4 The Norwegian Air Ambulance Foundation, Oslo, Norway; 5 Faculty of Health Sciences, Department for Prehospital Emergency Medicine, Oslo Metropolitan University, Oslo, Norway; 6 Air Ambulance department, Oslo University Hospital, Oslo, Norway; 7 Institute of Clinical Medicine, University of Oslo, Oslo, Norway; 8 Norwegian Directorate of Health, Norwegian Patient Registry, Oslo, Norway; 9 Faculty of Health and Welfare, Østfold University College, Fredrikstad, Norway; 10 Norwegian National Advisory Unit on Prehospital Emergency Medicine (NAKOS), Oslo University Hospital, Fredrikstad, Norway

**Keywords:** Prehospital care, Emergency medical services, Emergency calls, Emergency medical dispatch, Emergency medical communication center, Prehospital stroke management, Stroke pathway, Triage

## Abstract

**Objectives:**

In this study we aimed to explore EMCC triage of suspected and confirmed stroke patients to gain more knowledge about the initial phase of the acute stroke response chain. Accurate dispatch at the Emergency Medical Communication Center (EMCC) is crucial for optimal resource utilization in the prehospital service, and early identification of acute stroke is known to improve patient outcome.

**Materials and methods:**

We conducted a descriptive retrospective study based on data from the Emergency Department and EMCC records at a comprehensive stroke center in Oslo, Norway, during a six-month period (2019–2020). Patients dispatched with EMCC stroke criteria and/or discharged with a stroke diagnosis were included. We identified EMCC true positive, false positive and false negative stroke patients and estimated EMCC stroke sensitivity and positive predictive value (PPV). Furthermore, we analyzed prehospital time intervals and identified patient destinations to gain knowledge on ambulance services assessments.

**Results:**

We included 1298 patients. EMCC stroke sensitivity was 77% (95% CI: 72 − 82%), and PPV was 16% (95% CI: 14 − 18%). EMCC false negative stroke patients experienced an increased median prehospital delay of 11 min (*p* < 0.001). Upon arrival at the scene, 68% of the EMCC false negative patients were identified as suspected stroke cases by the ambulance services. Similarly, 68% of the false positive stroke patients were either referred to a GP, out-of-hours GP acute clinic, local hospitals or left at the scene by the ambulance services, indicating that no obvious stroke symptoms were identified by ambulance personnel upon arrival at the scene.

**Conclusions:**

This study reveals a high EMCC stroke sensitivity and an extensive number of false positive stroke dispatches. By comparing the assessments made by both the EMCC and the ambulance service, we have identified specific patient groups that should be the focus for future research efforts aimed at improving the sensitivity and specificity of stroke recognition in the EMCC.

**Supplementary Information:**

The online version contains supplementary material available at 10.1186/s12873-024-00962-7.

## Background

Outcome for patients with acute stroke depends on rapid diagnosis and treatment [1–4]. Emergency Medical Communication Centers (EMCC) is the first point of contact for the majority of patients with suspected stroke symptoms in Norway [5], and EMCC identification is crucial for efficient assessment to diagnostics and treatment in the chain of stroke survival [6, 7]. Early and precise EMCC recognition of stroke symptoms may result in high-priority dispatch of the ambulance service, increased awareness of stroke symptom among ambulance personnel, and pre-notification of stroke centers, reducing both pre- and in-hospital delay. Precise EMCC stroke recognition also enables utilization of specialized resources such as mobile stroke units (MSU) [8], and Helicopter Emergency Medical Services (HEMS) [9–11]. Reduced delays could improve outcome in both acute ischemic stroke (AIS) and intracerebral hemorrhage (ICH) [2, 12, 13]. 

EMCC assessments on emergency calls are challenging due to the dispatchers’ inability to visually observe or physically examine the patient. Nevertheless, stroke recognition in EMCC poses a particular challenge because stroke symptoms may be vague and unspecific, and because other medical conditions can mimic stroke symptoms [14–18]. Variations in EMCC stroke recognition rates is reported from 31 to 83% [16, 19–24]. and lack of EMCC stroke recognition (EMCC under-triage) may result in patient delay and lower treatment rates, consequently worsening outcome [2, 3, 14, 15]. On the other hand, medical conditions mimicking stroke, contributing to EMCC over-triage, could lead to overuse of limited pre- and in-hospital resources and under-prioritization of other medical emergencies [25, 26]. In this study we aimed to explore EMCC triage of suspected and confirmed stroke patients to gain more knowledge about the initial phase of the acute stroke chain and to identify potentials for improvement in EMCC stroke triage.

## Methods

### Study design and setting

We performed a descriptive retrospective study at Oslo University Hospital Ullevål (OUS), Norway, based on hospital emergency department and EMCC records during a six-month study period from, September 1st, 2019, until February 29th, 2020. OUS serves both as a local- and a comprehensive stroke center for a population of approximately 550 000 inhabitants with residential address in the municipality of Oslo. The EMCC Oslo serves a population of approximately 1.7 million inhabitants in both urban and rural parts of south-eastern Norway, including the Oslo metropolitan area, and handles approximately 250 000 emergency calls annually.

### EMS and prehospital response

All emergency calls to EMCCs in Norway are answered directly by specially trained EMCC nurses or paramedics using a Criteria-Based Dispatch (CBD) protocol. CBD protocols are decision triage support tools based on a patient’s signs and symptoms and require the dispatcher to have medical skills [27]. The current CBD dispatch protocol in use in Norwegian EMCCs is “The Norwegian Index for Emergency Medical Assistance” (Index) [28]. Index consists of a Start page and 39 different symptom-based criteria cards. The Start page focuses on obtaining critical information on the location of the event, patient state of consciousness, and initial assessment of whether the patient is suffering from an acute life-threatening illness or injury. Except for the Start page and criteria cards 1–3 (suspected cardiac arrests and unconsciousness), the remaining criteria cards each consists of listed criteria with corresponding responses and priorities, additional questions and supplemental information about symptoms and potential causes. Dispatch priorities are coded as red (acute and life-threatening conditions), yellow (urgent and potentially life- threatening conditions) and green (non-urgent conditions). After selecting the appropriate criteria card, the dispatchers assess listed criteria from top (the most acute criteria) to the bottom (non-urgent criteria) until a criteria is met. Stroke related dispatch criteria included in this study were identified from criteria card 27 (“Altered levels of consciousness/paralysis”) and criteria card 39 (“The eyes”) in the 3rd edition of the Norwegian Index (Supplemental material Illustration 1 and 2). Recent reports has shown a large variation in use of Index in assessment of acute stroke in Norwegian EMCCs [29]. EMCC operators have a relatively high self-reported use of Index as a support tool in dispatch decisions, however the study reports of with variations on both individual and EMCC level.

The ambulance service in Norway includes both ground- and boat ambulances staffed by paramedics or emergency medical technicians (EMT). It also includes HEMS and Search and Rescue (SAR) helicopters, staffed by prehospital critical care physicians. If patient symptoms are identified as suspected stroke symptoms by the EMCC dispatcher, the default response will be to dispatch the nearest ground ambulance with a stroke criteria and acute priority (lights and sirens). In remote areas, HEMS or SAR helicopters should be dispatched to reduce prehospital delay [11]. According to national legislation, 90% of all emergency calls to the emergency medical telephone number 113 must be answered within 10 s [30]. 

Patients with suspected stroke symptoms are routinely assessed by the ambulance service using the “Face– Arm– Speech– Time” test (FAST) [31] or prehospital National Institutes of Health Stroke Scale (NIHSS) due to the Paramedic Norwegian Acute Stroke Prehospital Project (ParaNASPP) [32], which involved five of the ambulance stations in Oslo during the study period. After clinical assessment, all patients with suspected stroke symptoms were consulted directly with the on-call stroke physician at OUS. If not accepted for hospitalization the patients were transported to their local hospital, general practitioner (GP) or out-of-hours GP acute clinic for diagnostic follow up.

### Data collection and calculations

Based on EMCC and hospital records we identified all patients who had an ambulance dispatched with one of the stroke criteria after initial EMCC assessments and/or was discharged from OUS with a main diagnosis of acute stroke. From the EMCC database we identified all dispatch records with stroke– and non-stroke criteria among patients discharged with stroke diagnosis. We extracted patient identity (name and Norwegian National Identity Number (11-digits)), dispatch criteria and priority, prehospital time stamps, and patient destination. All duplicates, false registrations, aborted or reallocated dispatches, were excluded to identify unique incidents. From the hospital records we identified all patients discharged with a main diagnosis of acute ischemic stroke (AIS) (I63.0-I63.9), intracerebral hemorrhage (ICH) (I61.0-I62.9), transient ischemic attack (TIA) (G45.8-G45.9), and other ICD-10 non-stroke diagnoses. We also extracted sex, age and referring body. We excluded patients referred from other hospitals, GP or out-of-hours GP acute clinic, patients admitted to local hospitals outside of Oslo, patients with missing EMCC records, and patients under the age of 18 years.

**Fig. 1:**
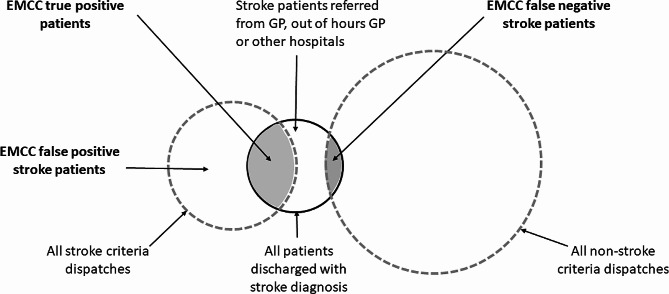
Patient inclusion and definitions. Patient groups are not proportional

Patients that had an ambulance dispatched with stroke criteria and who were discharged from OUS with a confirmed stroke diagnosis were defined as EMCC true positive. Patients that had an ambulance dispatched with stroke criteria who were not admitted to OUS or discharged with a non-stroke diagnosis, were defined as EMCC false positive. Patients discharged with a stroke diagnosis despite a non-stroke criteria dispatch, were defined as EMCC false negative. Definitions are illustrated in Fig. 1.

To identify subgroups of stroke patients with alternate prehospital pathways, such as patients initially referred to out-of-hours GP acute clinics by the ambulance service and thereafter admitted to OUS within 24 h after the initial emergency call, we matched hospital records with EMCC records, using the patients’ national identity number and incident date and time.

Destination for all patients were identified and categorized as; referred/admitted to OUS, referred to other hospitals, GP or out-of-hours GP acute clinics, or left on scene. Patients referred to OUS by the ambulance services without registered hospital admissions were defined as outpatients.

EMCC stroke sensitivity and positive predictive value (PPV) were calculated according to the definitions of EMCC true positive, false positive and false negative stroke patients. Due to the necessity of identifying and excluding all non-unique dispatches, patients referred from GP, out-of-hours GP or other hospitals, and patients admitted to hospitals outside of Oslo, the total number of true negative stroke patients was not identified. Consequently, EMCC negative predictive value (NPV) could not be calculated.

**Fig. 2:**
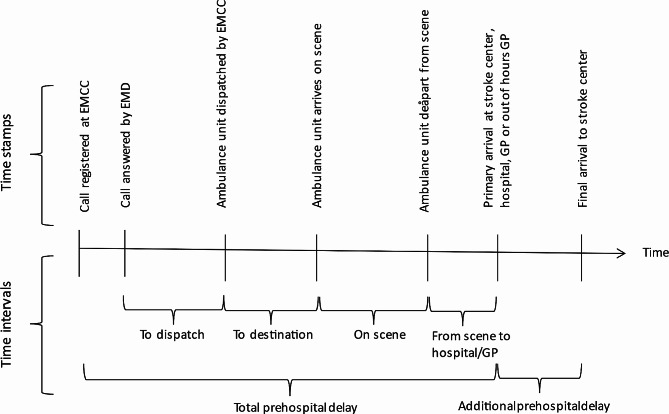
Prehospital timeline and time intervals. The Standards for Strengthening the Reporting of Observational Studies in Epidemiology (STROBE) guidelines were followed [33]

Prehospital time intervals were calculated based on EMCC electronic time stamps. Due to a lack of access to complete patient hospital records, patient delay (time interval from symptom onset until the initial emergency call to EMCC) was not included. Prehospital timeline and time interval definitions are presented in Fig. 2.

### Statistics

R version 3.6.1 was used to perform statistical calculations. Descriptive statistics are presented in numbers (%) and median with 25- and 75-percentiles. Time intervals are presented as median minutes and seconds (mm:ss) with 25- and 75-percentiles.

Groups were compared using Kruskal-Wallis Test. Wald test was used to calculate 95% confidence intervals for EMCC stroke sensitivity and positive predictive values (PPV).

*P*-values of < 0.05 were considered statistically significant.

### Ethics

The study is part of The Dispatch–Norwegian Acute Stroke Prehospital Project (Dispatch NASPP). The study has been performed according to the Helsinki convention [34]. Protocol for the Dispatch NASPP project was approved by the Regional Research Ethics Committee (REC) (ref. no. 2018/1909) and the local data protection officer at OUS (ref. no. 18/25,297).

After initial matching of data records from EMCC, ambulance, and hospital, all person identifiable data were replaced with a case number and the codebook was organized in Medinsight® Release 2.17.4.0. The identified dataset was handled in TSD - Service for Sensitive Data, at the University of Oslo, Norway. All EMCC nurses and paramedics employed in EMCC Oslo have been informed about the study.

## Results

**Fig. 3:**
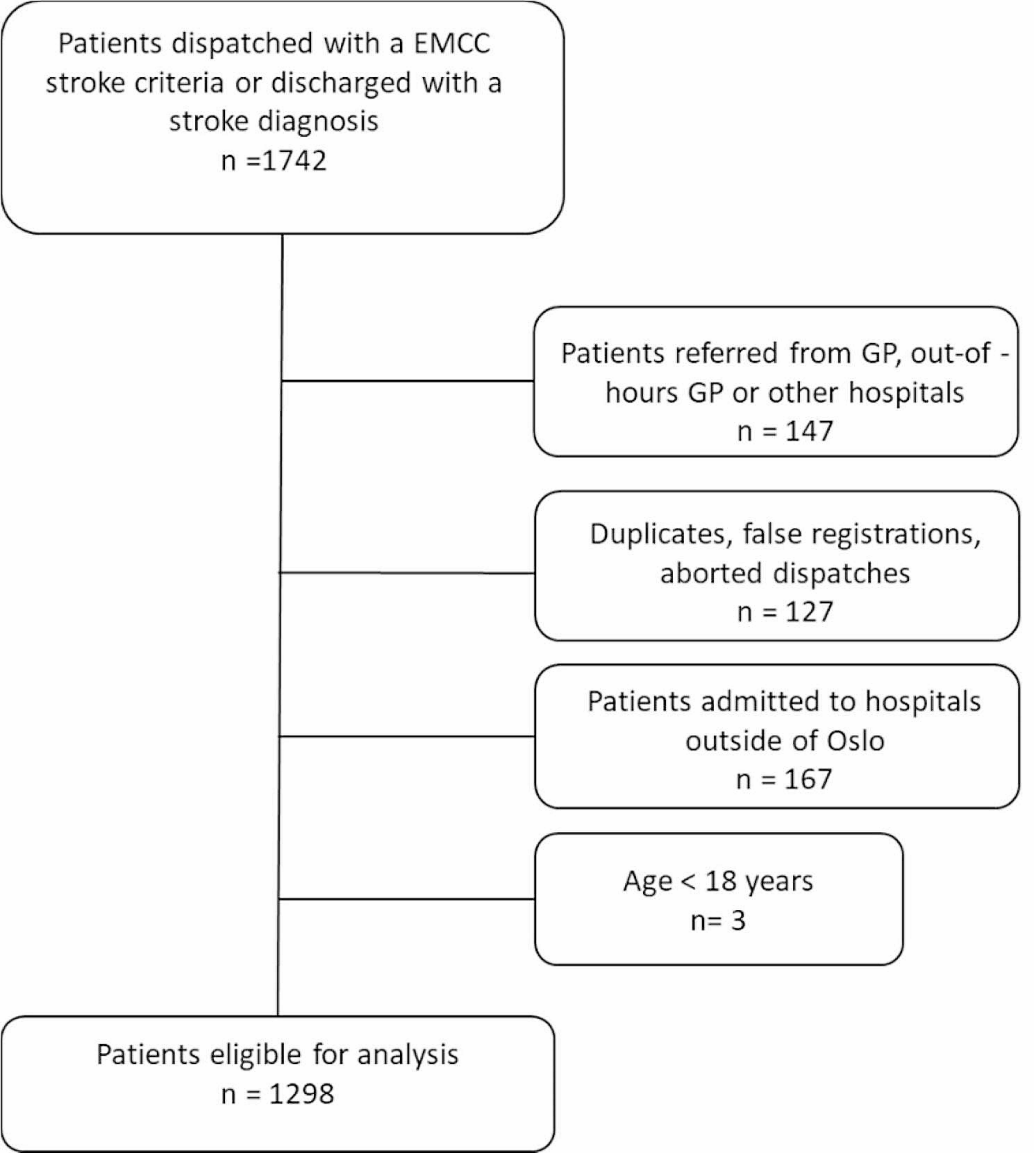
Flow chart study recruitment

We identified a total of 1742 patients dispatched with stroke criteria and/or discharged with a stroke diagnosis from OUS. We excluded 147 patients referred from GP, out-of-hours GP acute clinics or other hospitals and 127 EMCC duplicates, false registrations, aborted or reallocated dispatches. Furthermore, we excluded 167 patients admitted to hospitals outside of Oslo and three patients under the age of 18 years. As a result, 1298 patients were included in the analysis. Flow chart of study recruitment is presented in Fig. 3. Patient characteristics and main results are presented in Table 1 and supplemental materials, Tables 1 and 2.

**Table 1:** Patients characteristics

	All patients included *n* = 1298		True positive patients *n* = 201		False positive patients *n* = 1037		False negative patients *n* = 60		p =	
**Sex and age**										
Female n= (%)	688 (53)		99 (49)		560 (54)		29 (48)		0.354	
Age median (25 and 75 percentiles)	75 (61–85)		78 (69–87)		74 (60–85)		79 (63–87)		< 0.001	
**EMCC priority**										
Acute priority n= (%)	1239 (6)		199 (99)		1008 (97)		32 (53)		< 0.001	
Urgent priority n= (%)	57 (4)		2 (1)		29 (3)		26 (43)		< 0.001	
Non-urgent priority n= (%)	2 (0.2)		0 (0)		0 (0)		2 (3)		< 0.001	
**Verified stroke diagnoses at discharge (** ***n*** ** = 261)**										
Acute ischemic stroke (AIS) n= (%)			138 (68)		-		38 (63)		0.44	
Intracerebral haemorrhage (ICH) n= (%)			25 (12)		-		15 (25)		0.02	
Transient ischemic attack (TIA) n= (%)			38 (18)		-		7 (11)		0.19	
**Verified non-stroke diagnoses at discharge (** ***n*** ** = 286)**										
Diseases of the nervous system (G00– G99) n= (%)			-		51 (5)		-		NA	
Cerebrovascular diseases (except stroke) (I60-I69) n= (%)			-		19 (2)		-		NA	
Other symptoms and signs involving the nervous and musculoskeletal systems (R298) n= (%)			-		15 (2)		-		NA	
Symptoms and signs involving cognition, perception, emotional state and behavior (R40-R46) n= (%)			-		21 (2)		-		NA	
Symptoms and signs involving speech and voice (R47-R49) n= (%)			-		9 (0.9)		-		NA	
Other non-stroke diagnoses n= (%)			-		171 (17)		-		NA	

Age is presented as median with 25- and 75 percentiles. All other values are presented in numbers and (%). Proportions (%) are derived from the total number of true positive, false positive and false negative stroke patients. Groups were compared using Kruskal-Wallis Test. *P*-values of < 0.05 were considered statistically significant

### EMCC stroke sensitivity and positive predictive value

We found an overall EMCC stroke sensitivity of 77% (95% CI: 72– 82%) and a positive predictive value (PPV) of 16% (95% CI: 14– 18%). Cross-tabulation of dispatch criteria, final hospital discharge diagnosis, estimated EMCC stroke sensitivity and PPV are presented in supplemental materials Table 3.

### Time intervals

**Fig. 4:**
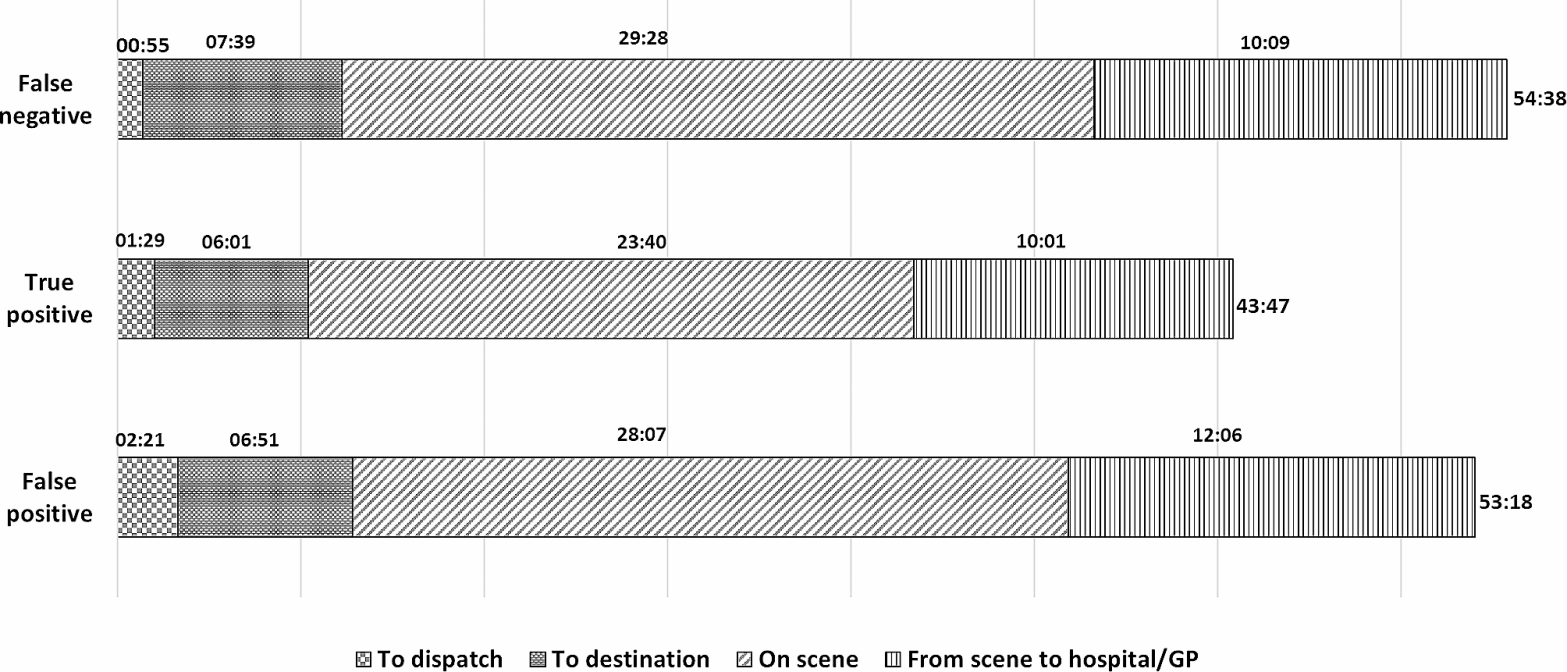
Prehospital time intervals. Prehospital time intervals are presented as median minutes and seconds (mm:ss). Total prehospital delay is presented on the end of each bar

The time interval from EMCC call answer to dispatch among true positive stroke patients was 01:29 (01:01–02:19) minutes. Total prehospital delay was 43:47 (35:59–53:35) minutes for patients admitted directly to OUS. Additional prehospital delay for the subgroup of patients initially referred to GP or out-of-hours GP acute clinics, was 111 (72–174) minutes. Prehospital time intervals for the main patient groups are illustrated in Fig. 4. Numbers and *p*-values are presented in supplemental materials Table 1.

Comparisons of EMCC false positive stroke patients and true positive stroke patients show a significantly increased time interval from call answer to dispatch (02:21 vs. 01:29 *p* < 0.001). Prehospital delay was not calculated for patients left on scene (*n* = 159). For the remaining false positive stroke patients (*n* = 878), we found a significant increased total prehospital delay (53:18 vs. 43:47 min *p* < 0.001).

Comparisons of EMCC false negative and true positive stroke patients is presented in Table 2 and show a significantly increased total prehospital delay (54:38 vs. 43:47 min *p* < 0.001), a significantly reduced call answer- to- dispatch time interval (00:55 vs. 01:29 *p* = 0.001), a significantly increased dispatch to destination time interval (07:39 vs. 06:01 *p* < 0.001) and a significantly increased on scene time interval (29:28 vs. 23.40 *p* = 0.001).

**Table 2:** Comparison of EMCC false negative and true positive stroke patients

	EMCC true positive *n* = 201	EMCC false negative *n* = 60	p =
Age	78.1 (69–87)	79.5 (63–87)	0.542
Female	99 (49)	29 (48)	0.90
Acute ischemic Stroke (AIS)	138 (69)	38 (63)	0.44
Intracerebral haemorrhage (ICH)	25 (12)	15 (25)	0.017
Transient ischemic attack (TIA)	38 (19)	7 (12)	0.19
EMCC acute priority	199 (99)	32 (53)	0.001
Time interval to dispatch	01:29 (01:01–02:19)	00:55 (00:21–03:55)	0.001
Time interval to destination	06:01 (04:45 − 08:03)	07:39 (06:22 − 12:09)	< 0.001
Time interval on scene	23:40 (18:02–29:29)	29:28 (20:25–41:42)	0.001
Time interval from scene to hospital/GP	10:01 (06:16 − 14:46)	10:09 (06:49 − 15:51)	0.492
Total prehospital delay	43:47 (35:59 − 53:25)	54:38 (40:08–69:48)	< 0.001

Age is presented as median with 25- and 75 percentiles. Sex, stroke diagnoses and priority are presented in numbers and (%). All time intervals are presented as median minutes and seconds with 25- and 75 percentiles. Groups were compared using Kruskal-Wallis Test. *P*-values of < 0.05 were considered statistically significant

### Patients with a suspected stroke at EMCC and discharged with a stroke diagnosis (true positive cases)

Among patients with a verified stroke diagnosis (*n* = 261), 201 (77%) patients had an ambulance dispatched according to EMCC stroke criteria and were defined as true positive stroke patients. 188 (94%) patients were admitted directly to the stroke center at OUS, while 13 (6%) patients initially were referred to GP or out-of-hours GP acute clinic by ambulance services and admitted to OUS within 24 h after the initial emergency call. Within this subgroup, 10 patients were diagnosed with AIS, none with ICH, and three with TIA. The proportion of women in this subgroup was 46% and median age was 70 (57–81) years.

The review of EMCC dispatch criteria showed that 174 (87%) of all true positive stroke patients were dispatched as “*A.27.03 Suddenly lop-sided (asymmetrical, irregular) in face*”, “A.27.04 *Sudden loss of strength in an arm or foot*”, or “A.27.05 *Sudden speech difficulties*” which corresponds to the stroke symptoms included in the Face-Arm-Speech-Time test (FAST). Among the remaining patients (*n* = 27), 23 (85%) patients were dispatched as “*A.27.06 Increasingly confused/drowsy, suspect stroke”*. There were no significant differences in either age, sex, or stroke diagnoses between these groups. Numbers and *p*-values are presented in supplemental material Table 2.

### Patients with a suspected stroke at EMCC and not diagnosed with stroke (false positive cases)

Among patients that had an ambulance dispatched with a stroke criteria, we found 1037 (84%) patients not diagnosed with stroke. As a result, they were defined as EMCC false positive stroke patients. 408 (39%) of all false positive stroke patients were referred to GP or out-of-hours GP acute clinics, 333 (32%) were referred to OUS, 159 (15%) were left on scene, and 137 (13%) were admitted to other local hospitals by the ambulance service. Flowchart describing the prehospital pathways of EMCC false positive stroke patients is presented in supplemental materials Fig. 3.

Compared to the group of true positive stroke patients, false positive stroke patients were younger, (74 vs. 78 years *p* < 0.001).

Among the group of false positive stroke patients admitted to OUS (*n* = 286), we identified 145 different non-stroke diagnoses, of which 115 (40%) patients were discharged with a non-stroke diagnosis related to symptoms from the nervous system (G00-G99, I60-I69 except stroke, R298, R40-R46 and R47-R49).

No patients left on scene by the ambulance service were referred to OUS within 24 h after the initial emergency call and discharged with a stroke diagnosis.

**Fig. 5:**
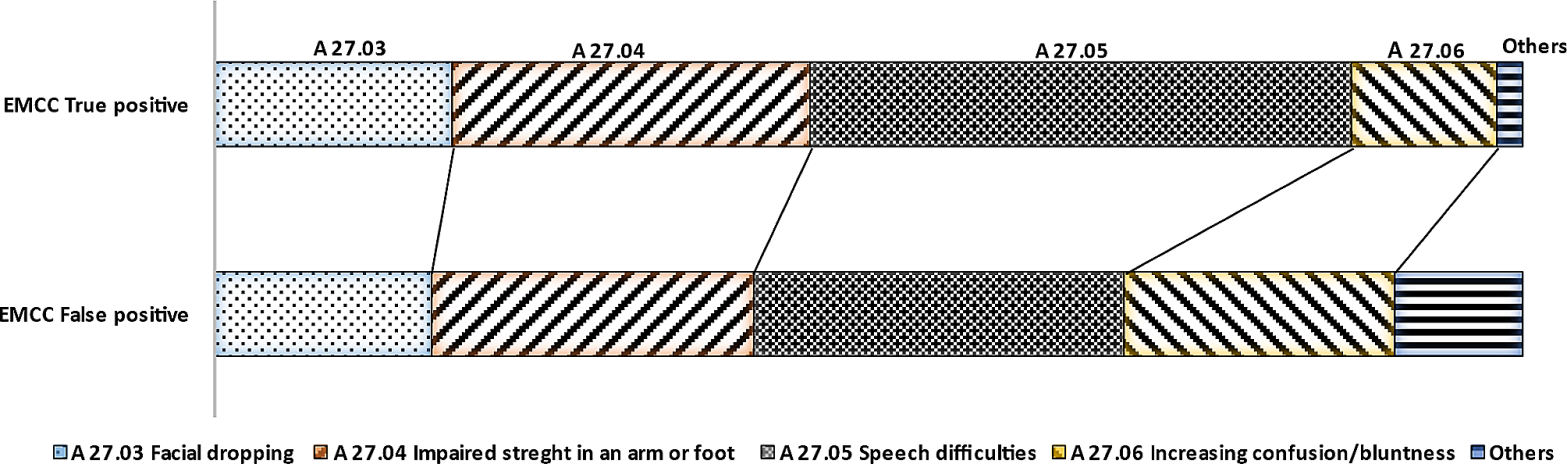
Proportions of stroke specific dispatch criteria among EMCC true positive and false positive patients

Compared to EMCC true positive stroke patients, false positive patients had a significant lower proportion of dispatch criteria *A.27.05 Sudden speech difficulties* (29% vs. 41% *p* < 0.001), a significant increased proportion of dispatch criteria *A.27.06 Increasingly confused/drowsy, suspect stroke* (21% vs. 11% *p* = 0.002), and *A.39.06 Sudden loss of vision in one eye* (27% vs. 0% *p* = 0.02). Proportions are illustrated in Fig. 5. Numbers and *p*-values are presented in supplemental materials Table 4.

### Patients with non-stroke suspicion at EMCC and discharged with a stroke diagnosis (false negative cases)

Among patients with a verified stroke diagnosis, we found that 60 (23%) patients were dispatched with a non-stroke criteria and defined as EMCC false negative stroke patients. 49 (82%) of all EMCC false negative stroke patients were brought directly to OUS by the ambulance services, of whom 41 out of 49 patients were referred as suspected stroke. 11 (18%) patients were initially referred to out of hours GP acute clinics or local hospitals by ambulance services and admitted to OUS within 24 h after the initial emergency call. Within this subgroup, seven out of 11 patients were diagnosed with AIS, two with ICH, and two with TIA. Median age in this small subgroup was 86 (73–88) years, the proportion of women was 82% and the additional prehospital delay was 112 (80–204) minutes.

Non-stroke criteria dispatches included 34 different dispatch criteria which could be categorized into the following main groups: “*Unclear problem*” (43%), “*Reduced consciousness*” (28%), “*Trauma/ wounds*” (10%), “*Chest pain*” (5%) and “*Headache*” (5%). All numbers and criteria codes are presented in supplemental materials Table 5.

Comparisons of EMCC false negative and true positive stroke patients show a significantly higher proportion of ICH diagnoses (25% vs. 12% *p* = 0.017), and a significantly lower proportion of acute priority dispatches (53% vs. 99% *p* = 0.001),

## Discussion

In this descriptive retrospective study, we found an EMCC stroke sensitivity of 77% and a positive predictive value (PPV) of 16%, indicating high rates of stroke recognition, but also very high rates of over-triage at dispatch for suspected acute stroke patients. We also found that stroke patients not identified by EMCC and under-triaged, experienced an additional prehospital delay of 11 min. These results highlight the important role of the EMCC in prehospital resource utilization and its potential impact on time to therapy for acute stroke patients.

An EMCC stroke sensitivity of 77% is among the highest reported regardless of whether using algorithm-based or criteria-based dispatch systems [14, 21–24, 35]. Even though there is no mathematical relationship between sensitivity and PPV, previous studies indicate a clear correlation between EMCC stroke sensitivity and PPV, indicating that increasing sensitivity is related to reduction in specificity and PPV, varying from a sensitivity rate of 53% and a corresponding PPV of 59%, to a sensitivity rate of 86% and a corresponding PPV of 20% [19–23, 35–38]. 

The most obvious explanation to the high stroke sensitivity found in this study is the exceptionally low PPV of 16%. Another likely explanation is that nearly 90% of all EMCC true positive stroke patients had pronounced stroke symptoms consistent with the Face-Arm-Speech– test [31]. This finding aligns with the results from other studies which suggest that patients with more pronounced stroke symptoms are more likely to contact EMCC rather than their own general practitioner or out-of-hours GP [39, 40]. This behavior could be attributed to public awareness campaigns that encourage individuals experiencing pronounced stroke symptoms to always call the EMCC promptly [41]..

Patients presenting subtle symptoms may not be identified as a suspected stroke [14–16], and often presupposes the gathering of more comprehensive patient information including medical history, medications, and possible risk factors. In this study, we found that nearly 50% of the false-negative stroke patients were dispatched with initial dispatch criteria of ‘Unclear problem’ and a “Non-acute priority”, indicating that no stroke symptoms or other life-threatening symptoms were identified by the dispatcher during the call. In the same population we found an EMCC call-to-dispatch time interval of 55 s, which indicates a very limited time frame for obtaining and processing relevant patient information before dispatch.

Interestingly there seems to be a correlation between dispatchers’ uncertainty about the cause of the patient’s symptoms, and the short time intervals they spend assessing dispatch criteria and priority. This could have various explanations. One potential explanation is the emphasis on rapid response outlined in the guidelines from the American Stroke Association, which recommends call-to-dispatch times of less than one minute [6]. Nevertheless, we also speculate that high workload, which is common in EMCCs, and the requirements to meet the national EMCC response time standards may have influenced the dispatcher’s assessments.

Patients who were not identified during the dispatch process, experienced an additional prehospital delay of 11 min. This delay can be attributed to several factors, including the extended ambulance response time which could be related to the high proportion of non-acute priority dispatches, but also the extended on-scene time interval.

Despite the absence of dispatch information regarding EMCC suspected stroke symptoms, it’s noteworthy that more than two-thirds of the false-negative stroke patients were ultimately identified as suspected stroke patients by the paramedics on scene. This highlights the critical role of paramedics in conducting clinical examinations and recognizing stroke symptoms. Consequently, the majority of EMCC false-negative stroke patients in this study were promptly transported directly to the stroke center, minimizing any additional prehospital delays.

The fact that ambulance personnel identified stroke symptoms in the vast majority of the EMCC false negative stroke patients upon their arrival, suggests that a larger number of patients could potentially have been recognized as stroke patients in EMCC. As a result, we argue that future research aimed at reducing EMCC under-triage should focus on this specific subgroup of EMCC false-negative stroke patients.

While some level of over-triage is acceptable to identify time-critical emergencies [25, 42], excessive EMCC dispatches with high priority can cause overuse of limited ambulance resources, under-prioritization of other medical emergencies and increased hospital admissions [25, 26, 42]. Excessive over-triage could also reduce the feasibility of dispatching Helicopter Emergency Medical Services (HEMS) to suspected stroke patients in remote areas [11–13]. 

In this study, we found 145 different non-stroke-related diagnoses among the false positive stroke patients admitted to the stroke center. This indicates that no specific diagnoses are systematically misinterpreted as strokes by EMCC dispatchers. Nevertheless, we found that 40% of these patients received diagnoses related to other neurological conditions that could be mistaken as stroke symptoms, defining them as stroke mimics. This finding is consistent with results from other studies showing that the prevalence of stroke mimics among patients admitted to emergency departments varies from 20 to 40% [17, 18, 43]. Even though patients with stroke mimics are often younger, have milder symptoms, and lack a history of vascular risk factors, it is rarely possible to predict whether patients with sudden-onset neurological symptoms have a stroke or stroke mimics without neuroimaging studies [17, 44]. As a result, we consider it less likely that this group of EMCC false-positive stroke patients could have been identified at the EMCC.

Another, and perhaps more intriguing, finding was that 68% of the false-positive patients were either transported to a GP, out-of-hours GP acute clinic, local hospital or left on the scene after assessment by the ambulance service. This finding suggests that most of the false-positive patients did not display any obvious stroke symptoms when the ambulance service arrived on scene. Although we know that the patient’s condition and symptoms can change over time, this finding leads to the question of whether EMCC dispatchers could have been more effective in ruling out stroke as a tentative diagnosis for a greater number of false-positive stroke patients. The results from this study cannot provide a definitive answer to that question. However, the results highlight the potential for further exploratory research with a more selective focus on EMCC false-positive stroke patients who were subsequently ruled out as stroke patients after assessment by the ambulance service.

### Strengths and limitations

The methodological approach of this study enables us to distinguish between EMCC and ambulance service assessments and to describe the different prehospital pathways of EMCC suspected and hospital verified stroke patients.

The external validity of this study is limited by the fact that the EMCC in question used a criteria-based dispatch protocol which is less common than algorithm-based dispatch systems. Nevertheless, results from published studies based on both algorithm-based and criteria-based dispatch systems reports substantial proportions of both EMCC false negative and false positive stroke patients [19–23, 35–38]. As a result, we argue that the results could be applicable for most EMCCs.

Studies comparing patients’ clinical symptoms at different time points should be interpreted with caution as the symptoms can change for better or worse. In this study we did not have access to hospital records from other hospitals than OUS. This may affect the results as some of the patients brought to other hospitals may have been diagnosed as stroke patients despite not being admitted to the stroke center at OUS. Furthermore, we did not assess stroke severity and outcome, nor did we systematically assess the pre-hospital consultations with in-hospital stroke physicians.

## Conclusions

Mis-triage at dispatch increases prehospital delay in acute stroke and negatively affects resource utilization. This study reports high EMCC stroke sensitivity, an extensive number of false positive stroke dispatches and a moderate number of false negative stroke dispatches. Furthermore, the study has identified specific patient groups that should be the focus for future research efforts aimed at improving the sensitivity and specificity of stroke recognition in the EMCC.

Our findings also emphasize the importance of the triage performed by ambulance personnel, which unlike EMCC dispatchers can visually observe and physically examine the patient and thereby limiting the negative effects of both EMCC over- and under-triage.

### Electronic supplementary material

Below is the link to the electronic supplementary material.

Supplementary Material 1

## Data Availability

Data for this study is stored pursuant to the security requirements stated by the Regional Committee for Medical and Health Research Ethics at Oslo University Hospital and at the Faculty of Medicine, University of Oslo. The unidentified datasets used for analysis for the current study are available through the corresponding author on reasonable request.

## Abbreviations

AIS: Acute Ischemic Stroke
AMIS: Acute Medical Information System
CBD: Criteria Based Dispatch
DIPS: Distributed Information and Patient data System
Dispatch NASPP: The Dispatch–Norwegian Acute Stroke Prehospital Project
ED: Emergency department
EMCC: Emergency Medical Communication Centre
EMD: Emergency Medical Dispatcher
EMS: Emergency Medical Services
EMT: Emergency Medical Technicians
FAST: Face Arm Speech Test
GP: General Practitioner
HEMS: Helicopter Emergency Medical Services
ICH: Intracerebral Hemorrhage
Index: The Norwegian Index for Emergency Medical Assistance
MSU: Mobile Stroke Unit
NIHSS: National Institutes of Health Stroke Scale
NPV: Negative Predictive Value
Norwegian Index: The Norwegian Index for Emergency Medical Assistance
OUS: Oslo University Hospital Ullevål
ParaNASPP: Paramedic Norwegian Acute Stroke Prehospital Project
PPV: Positive Predictive Value
REC: Regional Research Ethics Committee
SAR: Search and Rescue
TIA: Transient Ischemic Attack
TSD: Service for Sensitive Data
