# Supplementary material for: Telephone triage and dispatch of ambulances to patients with suspected and verified acute stroke - a descriptive study
Source: BMC Emerg Med. 2024 Mar 14;24:43. doi: 10.1186/s12873-024-00962-7 (PMC10941420; doi:10.1186/s12873-024-00962-7)
Supplement: Supplementary file 1 — Supplementary Material 1 [file 12873_2024_962_MOESM1_ESM.docx]

**SUPPLEMENTAL MATERIAL**

**Supplementary figure 1** The Norwegian Index for Emergency Medical Assistance, 3^rd^ edition, criteria card 27.


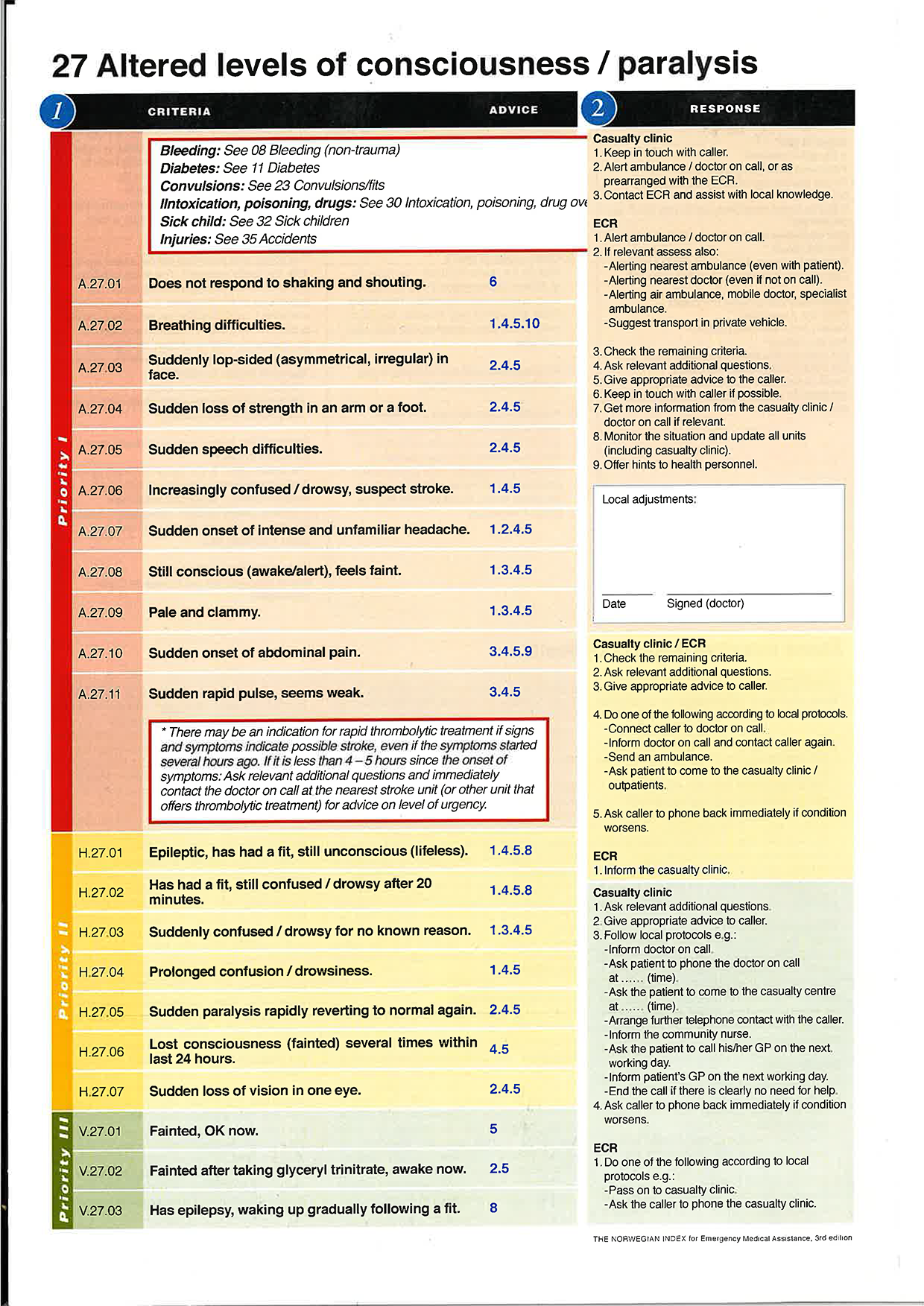


**Supplementary figure 2** The Norwegian Index for Emergency Medical Assistance, 3rd edition, criteria card 39.


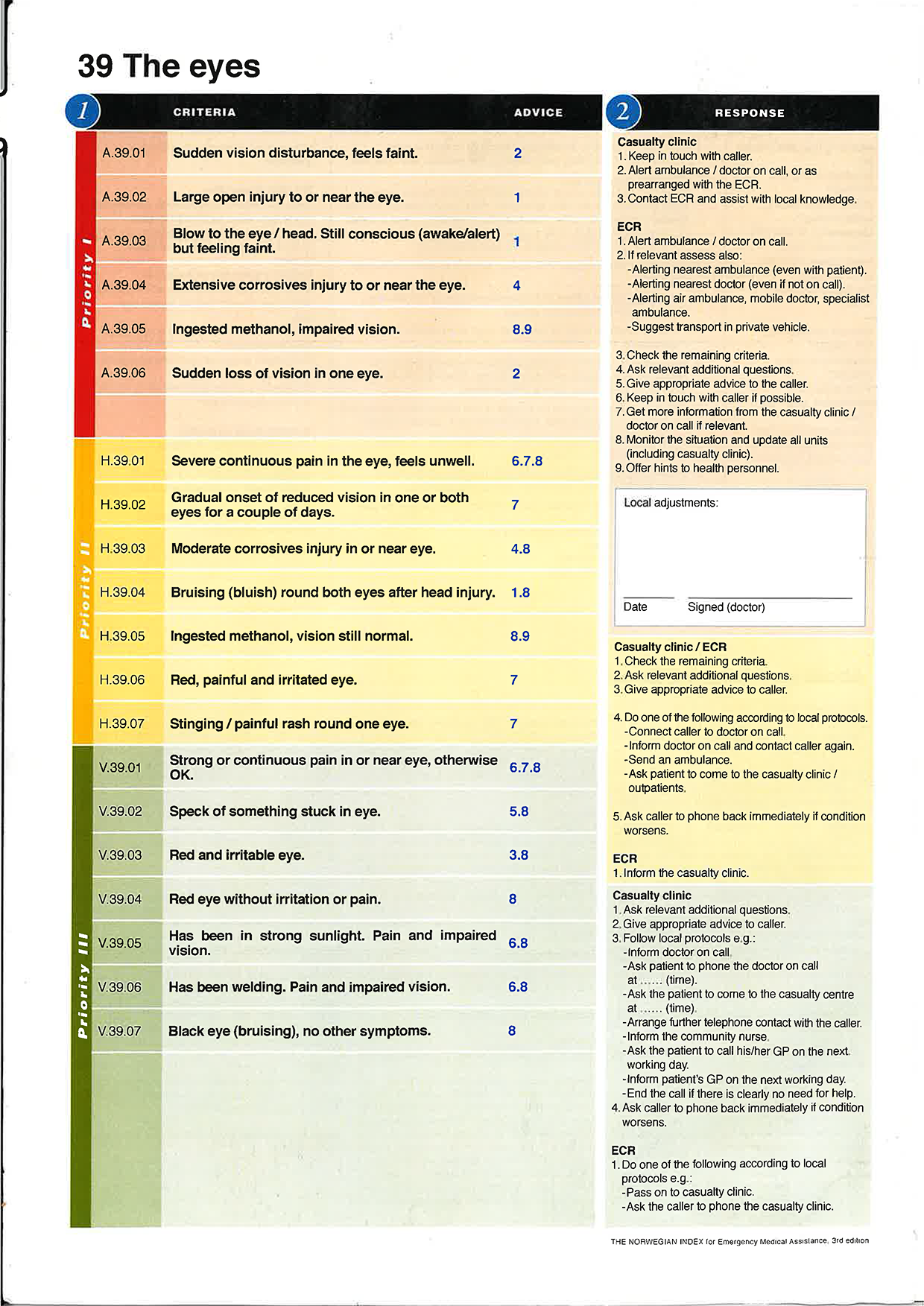


**Supplementary table 1** Comparison of prehospital time intervals among true positive, false positive and false negative stroke patients

|  | ***EMCC true positive patients***  ***n = 201*** | ***EMCC false positive patients***  ***n = 1037*** | ***EMCC false negative patients***  ***n = 60*** | ***p =*** |
| --- | --- | --- | --- | --- |
| Call to dispatch | 01:29  (01:01 – 02:19) | 02:21  (01:32-03:49) | 00:55  (00:21-03:55) | < 0.001 |
| Dispatch to destination | 06:01  (04:45-08:03) | 06:51  (05:15-09:03) | 07:39  (06:22-12:09) | < 0.001 |
| On scene median | 23:40  (18:02-29:29) | 28:07  (20:45-36:37) | 29:28  (20:25-41:42) | < 0.001 |
| From scene to hospital/GP | 10:01  (06:16 -14:46) | 12:06  (05:15-09:03) | 10:09  (06:49-15:51) | < 0.001 |
| Total prehospital delay | 43:47  (35:59-53:25) | 53:18  (42:35- 66:33) | 54:38  (40:08-69:48) | < 0.001 |
| Additional prehospital delay for the subgroups of patients initially referred to out of hours GP acute clinics | 110:43  (72:08- 173:57) |  | 111:53  (80:28-203:59) | 0.97 |

All time intervals are presented as median minutes and seconds (mm:ss) with 25- and 75-percentiles. Estimated total prehospital delay for false positive patients did not include patients left on scene (n=159) Groups were compared using Mann-Whitney U Test. P-values of < 0.05 were considered statistically significant.

**Supplementary table 2** Comparison of age, sex, and stroke diagnoses among main groups of true positive stroke patients.

| **Dispatch criteria** | ***A.27.03 - A.27.05***  ***n = 174*** | | ***A.27.06***  ***n= 23*** | ***p =*** |
| --- | --- | --- | --- | --- |
| Age | 78 (68 – 88) | | 75 (65 – 84) | 0.4076 |
| Female n= (%) | 84 (48) | | 12 (52) | 0.471 |
| Acute ischemic Stroke (AIS) n= (%) | | 120 (69) | 17 (74) | 0.75 |
| Intracerebral haemorrhage (ICH) n= (%) | | 22 (13) | 3 (13) | 0.92 |
| Transient ischemic attack (TIA) n= (%) | | 32 (18) | 3 (13) | 0.77 |

A.27.03 Suddenly lop-sided (asymmetrical, irregular) in face”, "A.27.04 Sudden loss of strength in an arm or foot", "A.27.05 Sudden speech difficulties, A.27.06 Increasingly confused/drowsy, suspect stroke.

Age is presented as median with 25- and 75-percentiles. Sex and stroke diagnoses are presented in numbers and (%). Groups were compared using Mann-Whitney U Test. P-values of < 0.05 were considered statistically significant.

**Supplementary table 3** Cross-tabulation of EMCC dispatch criteria and final discharge diagnosis, EMCC stroke sensitivity and positive predictive value (PPV)

|  |  | ***Final diagnose at discharge*** | | ***Total n, % (95 % CI)*** |
| --- | --- | --- | --- | --- |
|  |  | *Stroke diagnosis* | *Non-stroke diagnosis* |  |
| ***Dispatch criteria*** | *Stroke criteria* | 201 | 1037 | 1238 |
|  | *Non-stroke criteria* | 60 | NA | 60* |
|  | *Total* | 261 | 1037* | 1298 |
| ***EMCC stroke sensitivity*** | | | | 77.1 % (71.9 % – 82.1 %) |
| ***EMCC stroke positive predictive value (PPV)*** | | | | 16.3 % (14.2 % – 18.3 %) |

NA: The total number of unique non-stroke dispatches was not available. * Total number of patients with non-stroke dispatches and non-stroke diagnoses at discharge was not calculated

**Supplementary table 4** Comparison of dispatch criteria among true positive and false positive stroke patients

|  | *True positive patients*  *n = 201* | *False positive patients*  *n = 1037* | *p =* |
| --- | --- | --- | --- |
| A.27.03 Suddenly lop-sided (asymmetrical, irregular) in face n= (%) | 37 (18) | 174 (17) | 0.574 |
| A.27.04 Sudden loss of strength in an arm or a foot n= (%) | 54 (27) | 256 (25) | 0.51 |
| A.27.05 Sudden speech difficulties  n= (%) | 83 (41) | 299 (29) | < 0.001 |
| A.27.06 Increasingly confused/drowsy, suspect stroke n= (%) | 23 (11) | 217 (21) | 0.002 |
| A.27.07 Sudden onset of intense and unfamiliar headache n= (%) | 2 (1) | 29 (3) | 0.13 |
| A.39.01 Sudden visual disturbance, feels faint n= (%) | 0 (0) | 6 (0.6) | 0.27 |
| A.39.06 Sudden loss of vision in one eye  n= (%) | 0 (0) | 27 (3) | 0.02 |
| H.27.05 Sudden paralysis rapidly reverting to normal again n= (%) | 2 (1) | 26 (3) | 0.18 |
| H.27.07 Sudden loss of vision in one eye  n= (%) | 0 (0) | 3 (0.3) | 0.44 |

Values are presented in numbers and (%). Groups were compared using Mann-Whitney U Test. P-values of < 0.05 were considered statistically significant.

**Supplementary figure 3** Prehospital pathways for EMCC false positive stroke patients


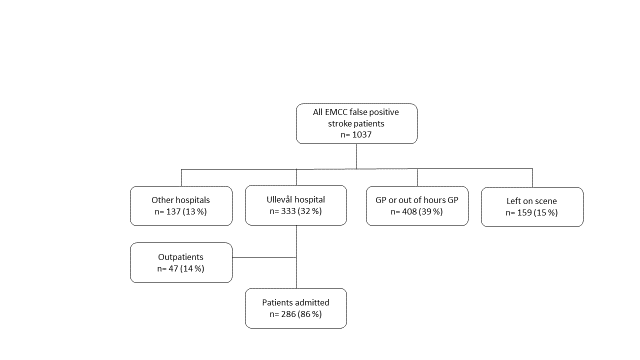


Patients presented in numbers (n=) and (%)

**Supplementary table 5** Dispatch criteria among false negative stroke patients

|  | | | *False negative patients*  *n = 60* | *p =* |
| --- | --- | --- | --- | --- |
| A.06.01 – A.06.11, H.06.01- H.06.12, V.06.03 (Unclear problem) n= (%) | | | 26 (43) | NA |
| A.01.03, A.27.01 - A27.09, H.27.03 - H.27.04 (Unconscious/reduced consciousness) n= (%) | | | 17 (28) | NA |
| H.33.02, H.33.04, H.35.05 (Trauma/wounds) n= (%) | | | 6 (10) | NA |
| A.10.07, H.10.01 (Chest pain) n= (%) | | | 3 (5) | NA |
| A.19.06 (Headache) n= (%) | | | 3 (5) | NA |
| A.07.03, A.11.01, A.23.2, A.29.02, H.25.01 (Others) n= (%) | | | 5 (8) | NA |
|  |  |  | | |

Values are presented in numbers and (%). Groups were compared using Mann-Whitney U Test. P-values of < 0.05 were considered statistically significant.
